# Potential of Bioactive Protein and Protein Hydrolysate from *Apis mellifera* Larvae as Cosmeceutical Active Ingredients for Anti-Skin Aging

**DOI:** 10.3390/ph17060679

**Published:** 2024-05-24

**Authors:** Paphawarin Thuraphan, Suphawan Suang, Anurak Bunrod, Watchara Kanjanakawinkul, Wantida Chaiyana

**Affiliations:** 1 Department of Pharmaceutical Sciences, Faculty of Pharmacy, Chiang Mai University, Chiang Mai 50200, Thailand; paphawarin_thu@cmu.ac.th (P.T.);; 2 Chulabhorn Royal Pharmaceutical Manufacturing Facilities by Chulabhorn Royal Academy, Chon Buri 20180, Thailand; anurak.bun@cra.ac.th (A.B.); watchara.kan@cra.ac.th (W.K.); 3 Center of Excellence in Pharmaceutical Nanotechnology, Faculty of Pharmacy, Chiang Mai University, Chiang Mai 50200, Thailand; 4 Multidisciplinary and Interdisciplinary School, Chiang Mai University, Chiang Mai 50200, Thailand

**Keywords:** *Apis mellifera*, alcalase, antioxidation, anti-ageing, cosmeceuticals, collagenase, hyaluronidase, lysine, protein hydrolysate, bee larvae

## Abstract

This study aimed to extract bioactive proteins and protein hydrolysates from *Apis mellifera* larvae and assess their potential application in cosmetics as well as their irritation properties. The larvae were defatted and extracted using various mediums, including DI water, along with 0.5 M aqueous solutions of sodium hydroxide, ascorbic acid, citric acid, and hydrochloric acid. Subsequently, the crude proteins were hydrolyzed using the Alcalase^®^ enzyme. All extracts underwent testing for antioxidant activities via the 2,2′-azino-bis (3-ethylbenzothiazoline-6-sulfonic acid) and Griess assays. Anti-aging properties were evaluated in terms of anti-collagenase and anti-hyaluronidase effects. Irritation potential was assessed using the hen’s egg chorioallantoic membrane (HET-CAM) test. The results revealed that the sodium hydroxide extraction showed promising outcomes in terms of yield, protein content, and effectiveness in inhibiting hyaluronidase, with the highest inhibition at 78.1 ± 1.5%, comparable to that of oleanolic acid. Conversely, crude protein extracted with ascorbic acid and its hydrolysate showed notable antioxidant and collagenase-inhibitory activities. Remarkably, their anti-collagenase effects were comparable to those of ascorbic acid and lysine. Additionally, it demonstrated safety upon testing with the CAM. In conclusion, the findings provided valuable insights into the utilization of *A. mellifera* larval proteins as active ingredients with a wide range of cosmeceutical applications, particularly due to their antioxidant, anti-aging, and low irritation properties, which hold significant promise for anti-skin wrinkles.

## 1. Introduction

*Apis mellifera*, recognized as economic insects that naturally inhabit hollow trees or branches, can also be managed in man-made nests, making honey collection more convenient. A large hive can house up to 50,000 bees, all descendants of the same queen bee, creating a cohesive family structure within the colony [1]. The queen bee, regarded as the ruler of the honeycomb kingdom, plays a vital role in sustaining the bee population by laying thousands of eggs at a time [2]. Bee products, such as honey, beeswax, propolis, and royal jelly, are currently consumed across a variety of industries, including foods, cosmetics, nutricosmetics, nutraceuticals, and cosmeceuticals [3,4]. These natural substances are known for their potential skincare benefits, including moisturization, antimicrobial properties, and anti-inflammatory effects [5,6].

Aside from these valuable products from *A. mellifera*, its larvae, after being roasted or grilled, have been commonly consumed as food by the local people of northern and northeastern Thailand and are readily available in the local market. On the other hand, *A. mellifera* larvae are often discarded as waste in the beekeeping industry, following the artificial larvae grafting process for royal jelly harvesting. While royal jelly is typically harvested for commercialization, leading to the larvae being considered waste, there is potential to explore and develop alternative applications for *A. mellifera* larvae alongside the royal jelly collection process. Despite this, there is currently a lack of research or reports regarding the utilization of *A. mellifera* larvae in the cosmetics, nutricosmetics, or cosmeceuticals industries. Exploring the cosmeceutical potential of *A. mellifera* larvae presents an intriguing and untapped area that merits further investigation since *A. mellifera* larvae has been reported to contain a rich nutritional profile, encompassing proteins, fatty acids, vitamins, and minerals such as calcium, magnesium, sodium, phosphorus, potassium, iron, and zinc [7]. As bees progress from the larval stage to the mature stage, their carbohydrate and fat contents decrease, while their protein content increases significantly, from 35.3% to 51.0% [8]. Previous research has also highlighted the presence of beneficial compounds in *A. mellifera* larvae, including vitamin C, coenzyme-Q10, α-tocopherol, retinol, and antioxidants, particularly phenolic compounds [9]. These bioactive constituents play a role in preventing degenerative changes induced by free radicals [9]. The abundant nutritional and bioactive composition of *A. mellifera* larvae, particularly peptides and proteins, make them a promising source of ingredients for various cosmetic and cosmeceutical applications. The utilization of *A. mellifera* larvae as a novel source of biologically active substances for cosmetics and cosmeceuticals would provide an opportunity to effectively utilize *A. mellifera* and its products. While honey, beeswax, propolis, and royal jelly are known for their beneficial characteristics and are already commercially valuable, exploring the potential of *A. mellifera* larvae could further increase the value of waste products, thereby offering economic opportunities for the beekeeping industry.

Nowadays, bioactive peptides are becoming increasingly popular in the beauty industry due to their potential to enhance skin health and appearance [10]. These small protein fragments could be divided into four categories, including signal, carrier, neurotransmitter inhibitor, and enzyme inhibitor, giving them a variety of biological effects, e.g., antioxidant, anti-aging, anti-inflammatory, and antibacterial capabilities, making them excellent cosmetic ingredients [10]. Certain peptides have been reported to trigger collagen and elastin synthesis, accelerate wound healing, promote fibroblast proliferation, mimic growth factors, and serve as agents for tensioning and tightening, resulting in enhanced skin health and appearance [11,12]. As consumers nowadays have begun to focus on health and beauty in a natural way by using natural products, whether they are medicinal plants or even animal products [13], products that contain natural raw materials that are chemical-free and environmentally friendly are growing rapidly, especially in the cosmetics and cosmeceutical industries [14]. Apart from natural cosmetic raw materials from plants, insects are another potential source of bioactive compounds for cosmetic applications.

Therefore, the present study aimed to extract proteins and protein hydrolysates from *A. mellifera* larvae and investigate their biological effects related to anti-skin aging, including antioxidant properties via radical scavenging, anti-inflammatory properties via nitric oxide inhibition, and anti-skin wrinkle properties via anti-collagenase and anti-hyaluronidase activities. In addition, the amino acid profile and irritancy properties of the *A. mellifera* larval extract were also evaluated.

## 2. Results and Discussion

### 2.1. Amino Acid Profiles of Dried and Defatted A. mellifera Larvae

Freeze-dried and defatted *A. mellifera* larvae were investigated for their amino acid profiles, as presented in Table 1**.** The findings indicated a consistent amino acid composition in both dried and defatted *A. mellifera* larvae, with lysine being the most abundant amino acid. The concentrations of lysine in freeze-dried and defatted *A. mellifera* larvae were 9.52 ± 0.18 and 8.61 ± 0.17% *w*/*w*, respectively. The results are consistent with prior research, which has consistently identified lysine as one of the predominant, essential amino acids in insects [15]. Lysine, found in the collagen α-peptide chain, can be hydroxylated to form hydroxylysine by the actions of hydroxylase enzymes, and this hydroxylation process is a characteristic modification of lysine residues in collagen, contributing to the stability and structure of collagen fibers [16]. Based on its role in collagen synthesis and its contribution to skin integrity, lysine could potentially have a positive impact on skin health and aging. Collagen is crucial for maintaining the skin’s firmness and elasticity, and lysine’s involvement in collagen cross-linking could help enhance skin structure [17]. Nevertheless, it is worth noting that in a prior study, glutamic acid was identified as the dominant amino acid, comprising 5.0% *w*/*w* or 18.6% of the total amino acids [8], which contrasts with the findings from the current study, where it was only around ~2% *w*/*w*. The likely explanation could be variations in sample sources or even inherent variability in the composition of the specific samples being studied.

### 2.2. Yields of Protein and Protein Hydrolysate from Apis mellifera Larvae

**Figure 1:**
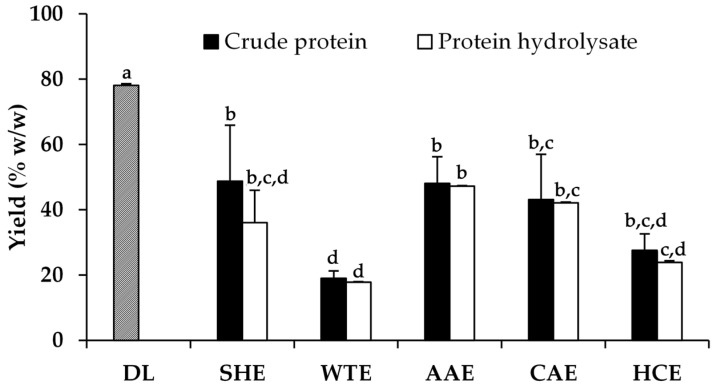
Yields of defatted *A. mellifera* larvae (DL) as well as crude protein and protein hydrolysate extracted using various media, including sodium hydroxide (SHE), DI water (WTE), ascorbic acid (AAE), citric acid (CAE), and hydrochloric acid (HCE). The data are expressed as the mean ± SD (*n* = 3). Lowercase letters (a–d) indicate significant differences between the *A. mellifera* larval extracts. The identical letters represent values that do not exhibit statistically significant differences. The data were analyzed using a one-way ANOVA, followed by Tukey’s post-hoc test (*p* < 0.05).

The defatted *A. mellifera* larvae, with a yield of 78.07 ± 0.48% *w*/*w* based on the dried weight of *A. mellifera* dried powder, were extracted using various mediums, including sodium hydroxide, DI water, ascorbic acid, citric acid, and hydrochloric acid, to yield the crude proteins, which were further hydrolyzed by the Alcalase^®^ enzyme to yield the protein hydrolysate. The yields of crude proteins and their protein hydrolysate presented in Figure 1 were calculated based on the dried weight of *A. mellifera* dried powder, with the statistical analysis results available in Tables S1 and S2. DI water was found to have the least potential in protein extraction since it yielded the smallest amount of crude protein of only 18.9 ± 2.3% *w*/*w* based on the dried weight of *A. mellifera* dried powder. In contrast, acids and bases assisted the crude protein extraction with no different yields, ranging from 43.1 ± 13.8% *w*/*w* to 48.7 ± 17.1% *w*/*w*, except for the extraction with hydrochloric acid (HCl). The likely explanations for the better extraction potential of both acidic and alkaline media are attributed to the isoelectric point (pI) values of most proteins, which typically fall within the range of 3.5 to 6.5 [18]. Generally, proteins have been considered less soluble in water at their respective pI values due to the electrically neutral state of protein molecules. As the pH of the DI water used for the extraction of *A. mellifera* larvae in the current study was 6.21, falling within the pI range of most proteins, the crude protein yield from the water extraction was hence found to be the lowest. On the other hand, the pH of the 0.5 M sodium hydroxide solution was 12.57, whereas the pH values of the 0.5 M ascorbic acid, 0.5 M citric acid, and 0.5 M hydrochloric acid solutions were 3.52, 2.63, and 1.46, respectively. These results were in line with the previous study, which reported that the solubility of the proteins contained in insects was found to be highly dependent on the pH during the extraction process, and the protein yields were higher in the acidic region at pH 2 and 3 [19]. Soetemans et al. (2019) also revealed that proteins can be separated from other non-soluble particles with the least protein loss at pH 2, and lactic acid at pH 2 was found to be more beneficial than at pH 3.3 [20]. Nevertheless, the current study found comparable yields of the crude protein extracted by ascorbic acid (pH = 3.52) and citric acid (pH = 2.63). But hydrochloric acid, which is traditionally used for the solubilization of proteins in the acidic region, yielded a lower amount of crude protein. The likely explanation could be attributed to the strong acidic properties of hydrochloric acid. The greater the deviation of the pH value from the pI values, the stronger the repulsion force within the protein tertiary structure, resulting in a more pronounced increase in the protein degradation rate [21]. Nevertheless, sodium hydroxide, despite being a strong alkali, still yielded a high content of crude proteins. One potential explanation is that the non-ionized heme’s interaction with the distal histidine in the strong alkaline solution could reduce its ability to bind by oxidation, thereby partially inhibiting the oxidation process of the strong alkali, resulting in a longer duration of protein degradation in the strong alkali solution compared to an acidic one [21]. Furthermore, a remarkable enhancement in protein solubility was noted under alkaline conditions, particularly at pH 9 and above [22]. As a protein’s solubility was lowest at its pI and increased as the pH deviated from the pI of 5 towards either acidic or alkaline conditions, Mishyna et al. (2019) discovered that the solubility of the *A. mellifera* protein at pH 3 was approximately comparable to that at pH 7, and it further increased with greater alkalinity [22]. However, the optimum condition of the isolation procedure depends on the proximate composition and the original amount of protein content in raw materials [23]. After each crude protein was hydrolyzed by the Alcalase^®^ enzyme, the findings exhibited no statistical difference between the yields of crude proteins and their protein hydrolysate. This can occur if the hydrolysis process is efficient and complete, meaning that all of the crude protein is successfully broken down into smaller peptides or individual amino acids without any loss or degradation. Nevertheless, relative to their crude proteins, the protein hydrolysate constituted 73.9%, 94.0%, 98.3%, 97.5%, and 86.4% *w*/*w*, respectively.

### 2.3. Protein Content of A. mellifera Larval Extracts

**Figure 2:**
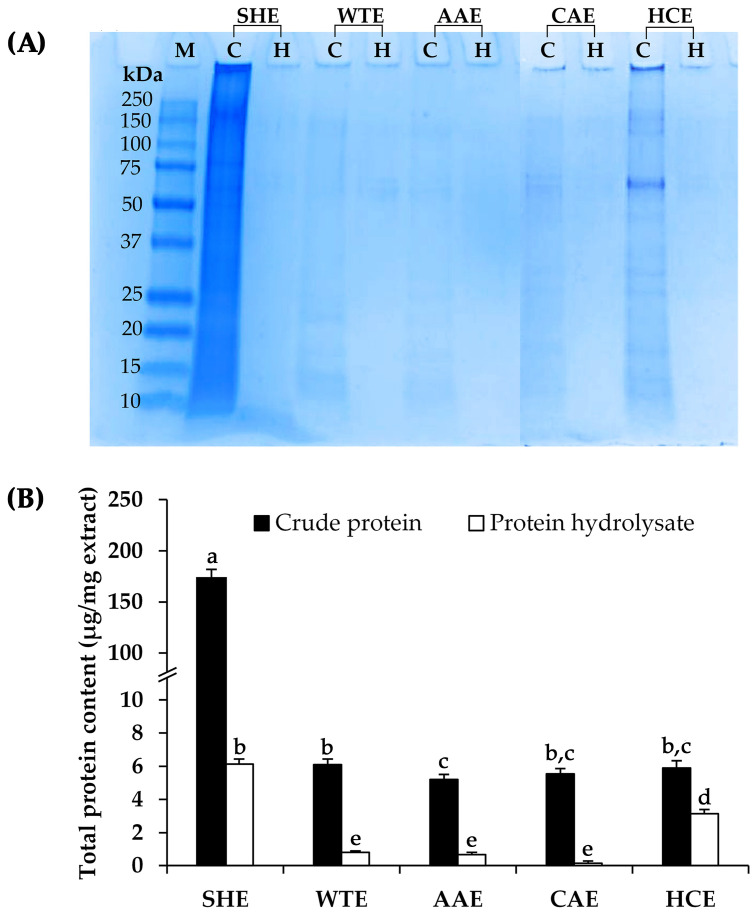
SDS-PAGE patterns of proteins (**A**) and total protein contents (**B**) of crude protein (C) and protein hydrolysate (H) extracted using various media, including sodium hydroxide (SHE), DI water (WTE), ascorbic acid (AAE), citric acid (CAE), and hydrochloric acid (HCE). M represents the protein molecular mass markers, ranging from 10 to 250 kDa. Lowercase letters (a–e) indicate significant differences between the *A. mellifera* larval extracts. The identical letters represent values that do not exhibit statistically significant differences. The data were analyzed using a one-way ANOVA, followed by Tukey’s post-hoc test (*p* < 0.05).

The crude proteins and protein hydrolysates from *A. mellifera* larvae were evaluated for their protein patterns by sodium dodecyl sulfate–polyacrylamide gel electrophoresis (SDS-PAGE), and the total protein content was also determined. Figure 2 illustrates that the SDS-PAGE patterns of proteins and the total protein contents of each *A. mellifera* larval extract were consistent. The crude protein extracted using a sodium hydroxide solution revealed a notably high protein content of 173.1 ± 8.8 µg/mg of extract, correlating effectively with the observed dark protein band on the SDS-PAGE. The previous study by Mishyna et al. (2019) similarly found that defatting and alkaline extractions were effective methods for enriching protein content in powder derived from *A. mellifera* because the solubility of proteins from *A. mellifera* was higher under alkaline conditions compared to acidic conditions [22]. The maximum solubility of *A. mellifera* protein was observed at pH 10, which was the highest pH value examined in the study conducted by Mishyna et al. (2019) [22]. Furthermore, Ryan et al. (1983) also indicated that the addition of sodium hydroxide resulted in varying degrees of protein solubilization, with the most severe treatment at pH 11.2 achieving a solubilization efficiency of 93% of the crude protein present in *A. mellifera* [24]. These results aligned with the current study, which revealed that the protein content of the *A. mellifera* larval extract was the highest when extracted using a 0.5 M sodium hydroxide solution at a pH of 12.57.

SDS-PAGE, a fundamental method for protein separation based on their charge and molecular structure [25], was used for analyzing the protein patterns of the *A. mellifera* larval extracts. The crude protein extract obtained from the sodium hydroxide extraction exhibited a prominent protein band, with the main bands observed at approximately 205 kDa and 80 kDa, as shown in Figure 2A. These protein bands were suggested to be vitellogenin and hexamerins, respectively [26]. Vitellogenin, a crucial protein generally considered female-specific, is primarily associated with female reproduction in various organisms, particularly *A. mellifera* [27]. However, substantial amounts of vitellogenin are synthesized in both female and male individuals during the larval stage, serving purposes beyond oocyte growth and energy provision to the embryo [28]. On the other hand, during larval development, hexamerins emerge as the primary products released by fat bodies into the insect hemolymph, exhibiting significantly higher abundance in *A. mellifera* larvae compared to *A. mellifera* adults, with expression levels exceeding 50-fold [26]. Remarkably, queens maintain hexamerin expression into adulthood at levels equivalent to larvae, thereby distinguishing them from other castes [26].

Nevertheless, protein bands were absent in all the protein hydrolysates, providing substantial evidence for the complete hydrolysis of crude proteins by the Alcalase^®^ enzyme into peptides. The reduction in the overall molecular weight of proteins to below 10 kDa of peptides or amino acids led to the disappearance of the protein band in SDS-PAGE. Previous research on peptide extraction from *A. mellifera* pupae indicated that the protein hydrolysate, generated through the Alcalase^®^ enzyme, comprised peptides with molecular weights ranging from 0.5 to 10 kDa, with a predominant range between 2 and 3 kDa [29]. These findings corresponded well with the yields of *A. mellifera* larval protein hydrolysates, ranging from 73.9% to 98.3%, indicating the successful breakdown of all crude proteins into smaller peptides or individual amino acids.

Aside from SDS-PAGE, the Bradford assay, which relies on the selective binding of a staining compound (Coomassie) via ionic interactions between sulfonic acid groups and positive amine groups on proteins [30,31], was used to assess the protein contents of *A. mellifera* larval extracts. Although there are several classical protocols alongside the Bradford assay for determining protein content, they also have their own limitations. The Biuret–Folin–Ciocalteu and Lowry methods are equally sensitive as the Bradford assay, but they are more laborious [25]. On the other hand, the bicinchoninic acid (BCA) assay relies on the interaction of proteins with Cu^2+^ ions, forming Cu^+^ complexes, which subsequently react with the BCA reagent, resulting in the development of a purple color [32]. However, it is noteworthy that proteins are not the sole compounds capable of reducing Cu^2+^ to Cu^+^. Various reducing agents, including ascorbic acid, have been reported to interfere with protein measurements in the BCA assay [33]. As ascorbic acid has the potential to reduce Cu^2+^ to Cu^+^, it can lead to a false positive, indicating an overestimation of the protein content [23].

Figure 2B illustrates the protein content derived from the Bradford assay of crude proteins extracted via various media. The statistical analysis results, available in Tables S3 and S4, showed no significant differences, except for the extraction using sodium hydroxide, which yielded a significantly highest protein content of 173.10 ± 8.81 µg/mg extract (*p* < 0.05). These findings were consistent with the protein patterns observed in SDS-PAGE. The explanation lies in the fact that the increase in protein solubility in alkaline conditions results from the unfolding of proteins and the formation of negatively charged amino acids due to deprotonation [34]. Similarly, the protein hydrolysate obtained from the crude protein extracted using alkali extraction exhibited the highest yield (6.13 ± 0.30 µg/mg extract) compared to the others. However, it was found that the protein hydrolysate contained considerably lower protein content. The likely explanation could be due to the limitation of the Bradford assay, which could not detect free amino acids and peptides with a molecular weight below 3 kDa [35]. The results were consistent with the data from SDS-PAGE, as there were almost no protein bands detected in the *A. mellifera* protein hydrolysate due to its low molecular mass. Despite the potential discrepancy in total protein content due to the small molecular weight of the protein hydrolysate, it is emphasized that the enzymatic hydrolysis process was successfully achieved.

### 2.4. Antioxidant Activities of A. mellifera Larval Extracts

**Figure 3:**
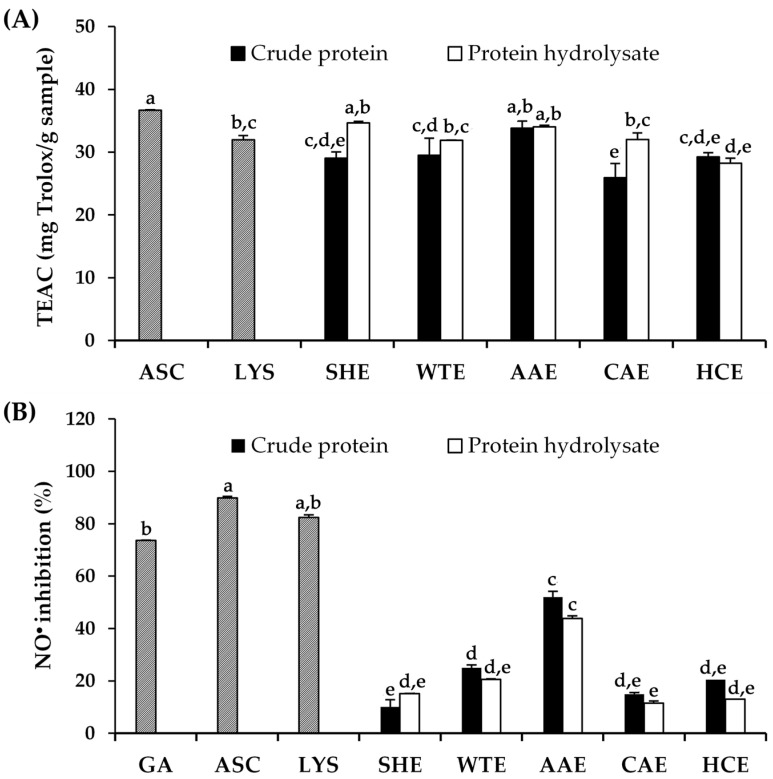
Antioxidant activities, in terms of Trolox equivalent antioxidant capacity (TEAC) (**A**) and nitric oxide (NO^•^) inhibition (**B**), of gallic acid (GA), ascorbic acid (ASC), lysine (LYS), and crude proteins and their hydrolysates extracted using various media, including sodium hydroxide (SHE), DI water (WTE), ascorbic acid (AAE), citric acid (CAE), and hydrochloric acid (HCE). Lowercase letters (a–e) indicate significant differences between the *A. mellifera* larval extracts. The identical letters represent values that do not exhibit statistically significant differences. The data were analyzed using a one-way ANOVA, followed by Tukey’s post-hoc test (*p* < 0.05).

As multiple reactions and mechanisms are known to be involved in the antioxidant process, the antioxidant activities of *A. mellifera* larval extracts were assessed using two different in vitro methods, including the ABTS assay and the Griess assay, as shown in Figure 3 and Table S5, with the statistical analysis results available in Tables S6–S9. The ABTS assay indicated the ability of the tested compounds to scavenge free radicals and was expressed as the Trolox equivalent antioxidant capacity (TEAC) value, while the Griess assay indicated the ability of the tested compounds to inhibit NO^•^, a reactive nitrogen species (RNS) related to oxidative stress and cellular damage [36,37]. The ABTS^•+^ inhibition of *A. mellifera* larval extracts ranged from 25.95 ± 2.25 to 33.81 ± 1.15 µg Trolox/mg sample, while that of their protein hydrolysate ranged from 28.23 ± 0.82 to 34.64 ± 0.27 µg Trolox/mg sample. The results revealed that all *A. mellifera* larval extracts exhibited comparable ABTS^•+^ radical scavenging activities, which was likely equivalent to that of lysine (31.97 ± 0.67 µg Trolox/mg sample), which was a major amino acid of *A. mellifera* larvae. On the other hand, only some extracts (crude protein and protein hydrolysate extracted by ascorbic acid as well as protein hydrolysate extracted by sodium hydroxide) demonstrated comparable ABTS^•+^ radical scavenging activity to that of ascorbic acid (36.65 ± 0.11 µg Trolox/mg sample), a well-known antioxidant. Therefore, these findings highlight the potential of *A. mellifera* larval protein extracts, particularly those extracted with ascorbic acid, as antioxidants, with applications in various industries, particularly cosmeceuticals and nutricosmetics, since antioxidants offer a scientific approach to improving skin health by protecting cells from oxidative damage and combating aging processes [38,39].

Although low-molecular-weight peptides are typically linked to enhanced antioxidant activity compared to larger peptides [40], the current study revealed that the crude protein and protein hydrolysate from *A. mellifera* larvae mostly exhibited no different effects. The probable rationale for this observation may lie in the notion that the essential attributes to the antioxidant efficacy of proteins or peptides are not solely constrained by their small molecular size and short chain length; rather, they encompass various factors, including the type, composition, sequence, and positional arrangement of amino acids within the peptide structure [41]. Fundamentally, the antioxidant potential has been associated with the steric, hydrogen bonding, electronic, and hydrophobic attributes of amino acids situated at the C- and N-terminals, which are governed by particular peptide configurations [42]. Crude proteins or their hydrolysates, comprising negatively charged acidic amino acids, e.g., glutamic acid and aspartic acid, have the potential to effectively scavenge free radicals via donating their excess electrons [43]. Furthermore, aromatic amino acids, e.g., tryptophan, phenylalanine, tyrosine, and histidine, have been associated with strong free radical scavenging activities via direct electron transfer [44].

Aside from the radical scavenging activities revealed by the ABTS assay, *A. mellifera* larval extracts were also investigated for their NO^•^ inhibitory activities. As NO^•^ can be biologically synthesized by endothelial cells, macrophages, and neurons, it serves as a vital chemical mediator, governing various physiological processes; however, elevated levels are associated with cytotoxic effects, particularly in aging [45]. Apart from oxidative stress, NO^•^ is also widely recognized as a major player in the inflammatory process. Following UV irradiation, NO^•^ plays a significant role in initiating melanogenesis, erythema, an immunosuppression and influencing various aspects of the inflammatory cascade, including its own expression and the recruitment of immune cells to the affected tissue [46,47]. Therefore, inhibiting NO^•^ could be used not only to evaluate antioxidant potential but also anti-inflammatory effects [48]. The NO^•^ inhibitory activities of protein extracts from *A. mellifera* larvae are shown in Figure 3B, with the statistical analysis results available in Tables S8 and S9. The NO^•^ inhibition of *A. mellifera* larval extracts ranged from 10.11 ± 4.23 to 51.98 ± 9.88%, while that of their protein hydrolysate ranged from 11.48 ± 2.66 to 43.84 ± 5.16%. The additional hydrolysis of *A. mellifera* larval crude extracts had no impact on NO^•^ inhibition. The findings indicated that crude protein extracted with ascorbic acid and its hydrolysate exhibited notable inhibitory effects against NO^•^, with the inhibition rates of 52.0 ± 9.9% and 43.8 ± 5.2%, respectively. The explanation may be attributed to the extraction of particular peptides or amino acids from the solvent with varying pH levels. Generally, the solubility of proteins is notably influenced by the pH and ionic strength of the solvent, leading to their dissolution in either acidic or alkaline solvents [34]. Proteins reach their lowest solubility at their pI, where no net charge is present. Nonetheless, proteins possess either a positive or negative net charge in various solvents, and the charged amino acids located on their surface engage with the ionic components of the solvent, facilitating protein dispersion and solubilization [34]. In acidic extraction, basic proteins are rendered soluble by neutralizing their positive charges, achieved through protonation of their amino groups under low pH conditions [34]. Conversely, alkaline extraction is preferable for acidic proteins, as the alkaline environment neutralizes their negative charges, primarily stemming from the deprotonation of their carboxyl groups at high pH levels [34]. For instance, basic amino acids like lysine, arginine, and histidine exhibit positive charges under acidic conditions but become neutralized in alkaline environments, while acidic amino acids such as glutamic acid and aspartic acid shift from neutral to negatively charged states as the pH increases. Additionally, alkaline conditions could break down the matrix, in which the proteins exist and making the protein more soluble in an aqueous extraction medium [49]. Consequently, acidic or alkaline extraction methods may yield distinct amino acid profiles in both crude proteins and their hydrolysates, as each method is tailored to the charge properties of the proteins being targeted. The current study suggested an ascorbic acid aqueous solution as the most suitable for extracting the protein with promising NO^•^ inhibition, which would be beneficial for both antioxidant and anti-inflammatory activities.

Therefore, these findings suggested that *A. mellifera* larvae are a novel natural source of antioxidants and anti-inflammatory agents. While other bee products are considered potential sources of natural antioxidants (e.g., propolis was found to possess antioxidant properties similar to those of the synthetic antioxidants butylated hydroxytoluene or ascorbic acid, while royal jelly was reported as a potent radical scavenger and exhibited strong reducing power) [50], the utilization of *A. mellifera* larvae, which is often discarded as waste in the beekeeping industry following the artificial larvae grafting process for royal jelly harvesting, would provide an opportunity to effectively utilize *A. mellifera*. In addition to the commercially valuable propolis and royal jelly, *A. mellifera* larvae could be another source of bioactive components, thereby increasing the economic value of beekeeping operations and offering new avenues for the development of cosmetics and cosmeceuticals.

### 2.5. Anti-Aging Activity of A. mellifera Protein Extracts

**Figure 4:**
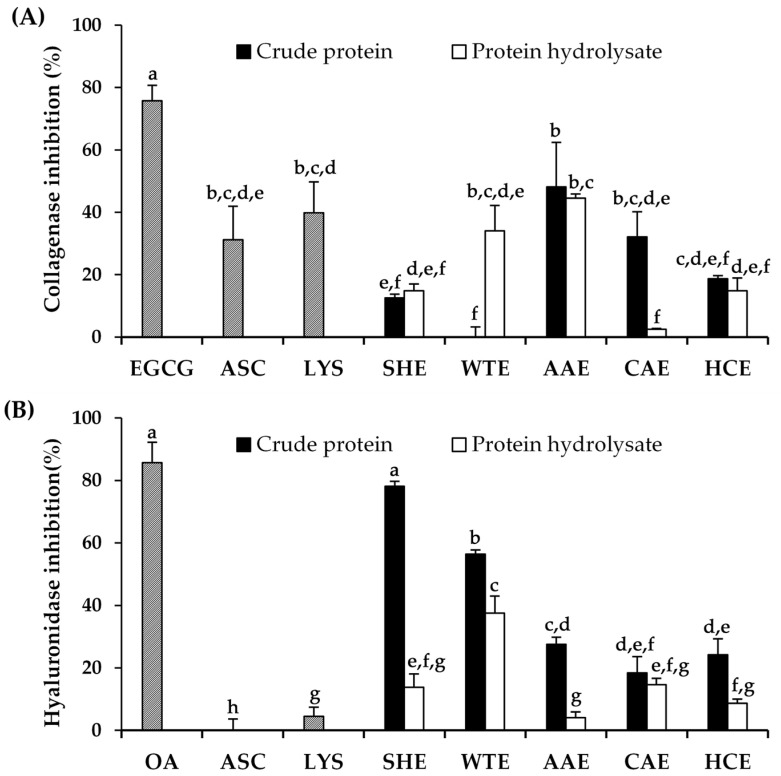
Anti-skin aging activities, in terms of collagenase inhibition (**A**) and hyaluronidase inhibition (**B**), of epigallocatechin gallate (EGCG), oleanolic acid (OA), lysine (LYS), and crude proteins and their hydrolysates extracted using various media, including sodium hydroxide (SHE), DI water (WTE), ascorbic acid (AAE), citric acid (CAE), and hydrochloric acid (HCE). Lowercase letters (a–h) indicate significant differences between the *A. mellifera* larval extracts. The identical letters represent values that do not exhibit statistically significant differences. The data were analyzed using a one-way ANOVA, followed by Tukey’s post-hoc test (*p* < 0.05).

The anti-aging activities of *A. mellifera* larval crude proteins and their protein hydrolysates were investigated in terms of their anti-collagenase and anti-hyaluronidase activities, as shown in Figure 4 and Table S10, with the statistical analysis results available in Tables S11–S14. The collagenase inhibition of *A. mellifera* larval crude extracts ranged from 0.00 ± 3.28 to 48.11 ± 14.26%. Notably, the crude protein extracted with ascorbic acid exhibited the most prominent anti-collagenase activity, whereas the aqueous extract showed no significant effect on collagenase inhibition. Additionally, the protein hydrolysate demonstrated comparable anti-collagenase activity to the crude extract, with the exception of the aqueous and citric acid extracts. This observation indicated the importance of the extraction method in determining the cosmeceutical efficacy of *A. mellifera* larval components. These findings indicated that the crude protein extracted with ascorbic acid and its hydrolysate exhibited notable inhibitory effects against collagenase, with the inhibition rates of 48.1 ± 14.3% and 44.5 ± 1.3%, respectively. Interestingly, their inhibitory effects were comparable to those of ascorbic acid (31.15 ± 10.81%) and lysine (39.86 ± 9.84%). The probable explanation may be ascribed to the capacity of acidic extractions to solubilize lysine, a basic protein, by neutralizing its positive charges through the protonation of lysine’s amino groups under low pH conditions [34]. On the other hand, hyaluronidase-mediated inhibition of *A. mellifera* larval crude extracts ranged from 18.39 ± 5.18 to 78.15 ± 1.55%. However, after hydrolysis by Alcalase^®^, the inhibitory activities significantly decreased from 4.09 ± 1.86 to 37.58 ± 5.37%. In contrast to the anti-collagenase-mediated inhibition, the crude protein extracted with sodium hydroxide displayed remarkable effectiveness in terms of its anti-hyaluronidase properties, with a significantly highest inhibitory activity of 78.1 ± 1.5%, which was equivalent to the inhibitory effects of oleanolic acid (85.66 ± 6.59%). The divergent findings in anti-collagenase and anti-hyaluronidase activities could be attributed to differences in the conformation of the active sites of collagenase and hyaluronidase. Despite the absence of a definitive conclusion regarding the suggested structure or functional group that corresponds to the active site of each enzyme, Pintus et al. (2023) proposed catechol groups at positions 5 or 6 of the coumarin scaffold, along with a bromine in the para or ortho positions of the 3-phenyl ring, as promising candidates for anti-hyaluronidase properties [51]. In contrast, several substitution patterns on the coumarin scaffold were suggested for anti-collagenase activities, i.e., two hydroxyl groups at positions 5 and 7, a bromine at the ortho, meta, or para positions of the 3-phenyl ring, and the presence of a hydroxyl group [51]. 

The findings from this research suggest that *A. mellifera* larval crude proteins have potential for anti-skin wrinkle effects, but the suitable extraction media need to align with the application purpose. As collagenase and hyaluronidase are enzymes that play pivotal roles in skin health, *A. mellifera* larval crude proteins, with their promising inhibitory activities against these enzymes, were appealing for use as anti-skin wrinkle active ingredients. Collagen, the primary structural protein in the dermis that provides the skin with resilience against stretching and contributes to its firmness, integrity, and flexibility, is generally broken down by the collagenase enzyme, potentially leading to premature skin aging [52,53]. Inhibitors of collagenase have the potential to prevent this degradation, thereby preserving the youthful appearance of the skin. On the other hand, hyaluronidase is the enzyme responsible for degrading hyaluronic acid, which is crucial for maintaining skin hydration, smoothness, and elasticity [54]. Inhibitors of hyaluronidase help regulate the balance between hyaluronic acid synthesis and breakdown, ensuring optimal skin moisture and smoothness [55].

### 2.6. Irritation Properties of A. mellifera Protein Extracts

**Figure 5:**
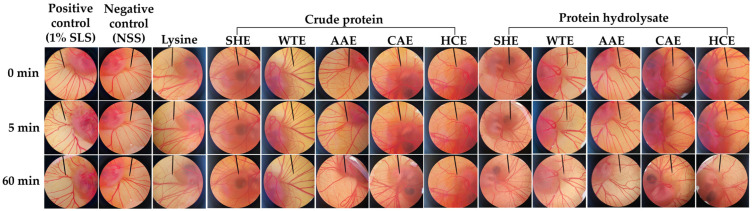
Photographs illustrating the chorioallantoic membrane (CAM) before (0 min) and after exposure to the tested compounds for 5 and 60 min. The 1% *w*/*v* sodium lauryl sulfate (SLS) was used as a positive control; normal saline solution (NSS) was used as a negative control; and lysine, a major amino acid detected in *A. mellifera* larvae, was also tested in a comparison with crude proteins and their hydrolysates extracted using various media, including sodium hydroxide (SHE), DI water (WTE), ascorbic acid (AAE), citric acid (CAE), and hydrochloric acid (HCE).

As a result of the notable antioxidant and anti-aging properties observed in the crude proteins and hydrolysates obtained from *A. mellifera*, they have been recommended as potentially beneficial active ingredients in cosmetic formulations. However, concerns have arisen regarding their potential to cause skin irritation. Consequently, the hen’s egg chorioallantoic membrane (HET-CAM) test, which does not necessitate ethical approval when the age of the hen embryo is less than half of the total incubation period, which is typically less than 10–11 days [56], was employed to assess the irritant potential of these *A. mellifera* larval extracts. As shown in Figure 5, the CAM displayed no signs of irritation after exposure to the negative control for both 5 and 60 min. In contrast, the positive control elicited an immediate reaction with the CAM, resulting in hemorrhage, followed by coagulation and vascular lysis. Consequently, all signs of irritation on the CAM were observed after 5 min of exposure and became more pronounced after 60 min. It is noteworthy that the 1% *w*/*v* sodium lauryl sulfate (SLS) induced severe irritation, as evident by an irritation (IS) score of 15.51 ± 0.64, as detailed in Table 2. This highlights the effectiveness of the standardized HET-CAM test for investigating the irritation potential of the tested compound, as evidenced by the absence of irritation in the negative and vehicle controls and the severe irritation induced by the positive control. On the other hand, the findings showed that neither the crude protein nor the protein hydrolysate, prepared using the Alcalase^®^ enzyme, caused any irritation to the CAM at the tested concentration of 10 mg/mL. However, vascular hemorrhage and lysis were observed in the CAM after 60 min of exposure to all of the protein hydrolysates. The residue of the Alcalase^®^ enzyme could be a reason for these irritation signs. However, it is crucial to emphasize that the irritation scores were derived from observations conducted over a 5-min timeframe [57]. Therefore, it can be concluded that *A. mellifera* larval crude proteins are generally safe and can be considered for use as ingredients in cosmetics without causing irritation to consumers.

## 3. Materials and Methods

### 3.1. Chemical Materials

L-ascorbic acid and citric acid used for crude protein extraction were cosmetic-grade products purchased from Chanjao Longevity Co., Ltd. (Bangkok, Thailand), whereas sodium hydroxide and hydrochloric acid were analytical-grade products purchased from Sigma-Aldrich (St. Louis, MO, USA) and RCI Labscan Co., Ltd. (Bangkok, Thailand), respectively. The Alcalase^®^ enzyme from *Bacillus licheniformis* used for protein hydrolysis was purchased from Merck KGaA (Darmstadt, Germany). Bovine serum albumin (BSA), 6-hydroxy-2,5,7,8-tetramethylchroman-2-carboxylic acid (Trolox), 2,2′-azino-bis (3-ethylbenzthia zoline-6-sulphonic acid) (ABTS), Griess reagent (modified), hyaluronic acid, phosphoric acid, sodium nitroprusside (SNP), L-tyrosine, 3,4-dihydroxy-L-phenylalanine (L-DOPA), sodium chloride (NaCl), SLS, Tris base, tyrosinase from mushroom (EC 1.14.18.1), hyaluronidase from bovine testes (EC 3.2.1.35), N-(3-[2-Furyl]acryloyl)-Leu-Gly-Pro-Ala (FALGPA), elastase from porcine pancreas (EC 3.4.21.36), collagenase from *Clostridium histolyticum* (EC 232-582-9), N-succinyl-Ala-Ala-Ala-p-nitroanilide (AAAPVN), sodium acetate, sodium phosphate dibasic dihydrate (Na_2_HPO_4_·2H_2_O), sodium phosphate monobasic dihydrate (NaH_2_PO_4_·2H_2_O), potassium chloride (KCl), potassium dihydrogen phosphate (KH_2_PO_4_), sodium hydroxide (NaOH), epigallocatechin gallate (EGCG), oleanolic acid, and L-ascorbic acid were all analytical-grade chemicals purchased from Sigma-Aldrich (St. Louis, MO, USA). Dulbecco’s Modified Eagle Medium (DMEM), penicillin/streptomycin, fetal bovine serum (FBS), and glucose were purchased from Invitrogen™ (Grand Island, NY, USA). Ferrous sulfate (FeSO_4_), ferric chloride (FeCl_3_), potassium persulfate (K_2_S_2_O_8_), sodium chloride (NaCl), calcium chloride (CaCl_2_), and calcium chloride (CaCl_2_) were analytical-grade chemicals purchased from Fisher Chemicals (Loughborough, UK). Ethanol, dimethyl sulfoxide (DMSO), 37% hydrochloric acid (HCl), and acetic acid were analytical-grade solvents purchased from RCI Labscan Co., Ltd. (Bangkok, Thailand). SDS buffers, reagents, and acrylamide for protein electrophoresis, along with laemmli buffer and Coomassie blue (dye reagents) for protein content determination, were purchased from Bio-Rad (Richmond, CA, USA).

### 3.2. A. mellifera Larvae Material

The frozen larvae of *A. mellifera* were obtained from the Faculty of Agro-Industry, Chiang Mai University, Chiang Mai, Thailand. Consequently, the *A. mellifera* larvae were subsequently subjected to freeze-drying (LyoQuest laboratory freeze dryer, Telstar, Terrassa, Spain). After the *A. mellifera* larvae were ground into a powder, they were kept in a sealed aluminum foil bag at room temperature for further use.

### 3.3. Defatting Process of A. mellifera Larvae

The dried *A. mellifera* larvae powder was defatted using hexane at a weight-to-volume ratio of 1:5. After the mixture was shaken for 1 h, the hexane was removed using filter fabric (local market, Chiang Mai, Thailand) [19]. The residue of *A. mellifera* larvae was then collected and dried under a laboratory chemical fume hood (EFD-4B8 Esco Frontier™ Duo Fume Hood, Esco Lifesciences (Thailand) Co., Ltd., Bangkok, Thailand) to facilitate the complete evaporation of the remaining hexane [58]. The defatted *A. mellifera* larvae were stored in a tightly closed container at room temperature until further use.

### 3.4. Amino Acid Profile Determination

The amino acid profiles of *A. mellifera* larvae and *A. mellifera* larvae powder were determined by Central Lab Thai, Chiang Mai, Thailand, using AOAC official methods 994.12 and 988.15 [59,60]. For the analysis of all amino acids except tryptophan, the samples underwent performic acid oxidation. Amino acids were hydrolyzed using 6 M HCl. The resulting hydrolysates were diluted with sodium citrate buffer and neutralized, with the pH adjusted to 2.20 before examination. For the analysis of tryptophan, a different method was employed. The samples were hydrolyzed using 4.2 M NaOH under vacuum, and pH adjustment and clarification followed. Individual amino acids were detected using a GC model 6890 N (Agilent, Waldbronn, Germany) with an MS detection model 5973 inert (Agilent Technologies, CA, USA), employing a Zebron ZB-AAA column (10 m × 0.25 mm, 0.25 µm film thickness, Phenomenex, Torrance, CA, USA).

### 3.5. Crude Protein Extraction from A. mellifera Larvae

The defatted *A. mellifera* larvae powder was extracted using various extraction media, including deionized (DI) water as well as 0.5 M aqueous solutions of ascorbic acid, citric acid, hydrochloric acid, and sodium hydroxide. The extraction was conducted following the method of Amarender et al. (2020) with some modifications [19] at a weight-to-volume ratio of 1:6 using an orbital shaker (Innova™2100 Eppendorf, Hamburg, Germany) for 1 h. The residues of *A. mellifera* larvae were eliminated using filter fabric, following which the resulting filtrate was centrifuged at 10,000× *g* at 4 °C for 20 min using a Hanil MF80 Benchtop Centrifuge (Hanil Science Industrial Co., Ltd., Incheon, Republic of Korea). The supernatant was collected and neutralized to pH 7.0 and subjected to freeze-drying using a freeze dryer (LyoQuest laboratory freeze dryer, Telstar, Terrassa, Spain). The crude proteins were obtained, and their yields were calculated by the following equation:

Yield of crude protein (%) = (A/B) × 100,(1)

where A is the weight of each crude protein (g) and B is the weight of defatted *A. mellifera* larvae (g).

### 3.6. Protein Hydrolysis by the Alcalase^®^ Enzyme

Each *A. mellifera* larval crude protein was hydrolyzed by the Alcalase^®^ enzyme, following the method of Li et al. (2005) [61]. The hydrolysis condition was set at pH 8.0 and a temperature of 55 °C. After adding 2.4 units/g of the Alcalase^®^ enzyme to the crude protein in a ratio of 2:100, the hydrolysis process was conducted for 10 h while maintaining a pH of 8.0 using a 1.0 M NaOH solution. Subsequently, the resulting mixture was immediately heated in a water bath set at 100 °C for 10 min to inactivate the Alcalase^®^ enzyme. After cooling down to room temperature, the resulting mixture was filtered through a Whatman^®^ No. 1 filter paper (Cytiva Life Sciences, Marlborough, MA, USA). The filtrate was collected and subsequently freeze-dried, resulting in a protein hydrolysate. The yields of *A. mellifera* larval protein hydrolysates were calculated by the following equation:

Yield of protein hydrolysate (%) = (A/B) × 100,(2)

where A is the weight of each protein hydrolysate (g) and B is the weight of defatted *A. mellifera* larvae (g).

### 3.7. Total Protein Content Determination by the Bradford Assay

*A. mellifera* larval crude proteins and their protein hydrolysates were investigated for their total proteins content using the Bradford assay [31]. The working reagent was prepared by mixing 1 part of a dye solution with 4 parts DI water. After that, 20 µL of each sample was combined with 180 µL of the working reagent and incubated at room temperature for 5 min. Following the incubation, the resulting mixtures were measured for their absorbance at 595 nm using a microplate reader (BMG Labtech, Ortenberg, Germany). The experiments were conducted in triplicate, and the protein content was calculated using the bovine serum albumin standard curve.

### 3.8. Molecular Weight Distribution Determination by SDS-PAGE

*A. mellifera* larval crude proteins and their protein hydrolysates were examined for their molecular weight distribution using SDS–PAGE [62,63]. In brief, each sample was dissolved in DI water and mixed with Laemmli buffer in a volume ratio of 3:1. Subsequently, the resulting mixtures were loaded into individual wells of a 12% SDS-PAGE gel. Electrophoresis was conducted at 100 V for 1 h or until the bands reached the designated line. Following the separation, the gel was fixed using a solution of 10% *v*/*v* acetic acid and 40% *v*/*v* methanol. After the 30-min fixing process, the gel was stained with Coomassie blue for 1 h. Subsequently, the gel underwent destaining with 10% *v*/*v* acetic acid three times, with each interval lasting 15 min, or until the gel achieved clarity. The protein bands were analyzed using the Gel Doc^TM^ EZ imager (Bio-Rad Laboratories, Hercules, CA, USA).

### 3.9. Antioxidant Activity Determination 

#### 3.9.1. 2,2′-Azino-bis (3-ethylbenzthia zoline-6-sulphonic Acid) (ABTS) Assay

*A. mellifera* larval crude proteins and their protein hydrolysates were investigated for their scavenging ability against ABTS^•+^ radicals using the ABTS assay, which was modified from the method of Miller and Rice-Evans (1996) [64]. A stock solution of ABTS^•+^ radicals was prepared by mixing a 7 µM aqueous solution of ABTS with a 2.45 mM K_2_S_2_O_8_ solution. The resulting solution was stored in the dark for 16 h, diluted with DI water, and mixed with 20 µL of a 1 mg/mL protein extract aqueous solution for 5 min at room temperature. The mixtures containing the *A. mellifera* larval crude proteins at a final concentration of 0.1 mg/mL were measured for their absorbance at 750 nm by a microplate reader (BMG Labtech, Ortenberg, Germany). The experiments were conducted in triplicate. The results were calculated and presented as Trolox equivalent antioxidation capacity (TEAC) values, which was calculated using the following equation:

TEAC (µg Trolox/mg extract) = (A − 0.8571)/0.0225,(3)

where A is the absorbance of each sample tested in the ABTS assay. Ascorbic acid was used as a positive control.

#### 3.9.2. Griess Assay

*A. mellifera* larval crude proteins and their protein hydrolysates were investigated for NO^•^ inhibition using the Griess assay, which was modified from the method of Sumanont et al. (2004) [37]. Briefly, 80 µL of 10 mM SNP in PBS pH 7.4 was mixed with 20 µL of a sample solution and incubated for 150 min. The modified Griess reagent, which contains 1% *w*/*w* sulfanilamide and 0.1% *w*/*w* NED in 2% *w*/*w* H_3_PO_4_, were added in a 1:1 ratio and incubated for 10 min at room temperature in the dark. The mixtures containing the *A. mellifera* larval crude proteins at a final concentration of 0.1 mg/mL were measured for their absorbance at 540 nm by a microplate reader (BMG Labtech, Ortenberg, Germany). The experiment was conducted in triplicate. The inhibition of NO^•^-free radicals was calculated using the following equation:

NO^•^ inhibition (%) = 1 − (A/B) × 100, (4)

where A and B are the absorbance values of the mixture with and without the sample, respectively. Gallic acid was used as a positive control.

### 3.10. Anti-Aging Activity Determination

#### 3.10.1. Inhibition of the Collagenase Enzyme 

*A. mellifera* larval crude proteins and their protein hydrolysates were investigated for their collagenase inhibitory activity based on the study of Chaiyana et al. (2019) [36]. Briefly, samples were incubated with 0.8 mg/mL collagenase in 50 mM Tricine buffer (pH 7.5, with 400 mM NaCl and 10 mM CaCl_2_) for 15 min. After that, 2 mM of the synthetic substrate N-[3-(2-furyl) acryloyl]-Leu-Gly-Pro-Ala (FALGPA) was added to the mixtures. After adding the substrate, the resulting mixtures containing the *A. mellifera* larval crude proteins at a final concentration of 0.1 mg/mL were measured for their absorbance at 340 nm immediately and continued for 20 min using a microplate reader (BMG Labtech, Ortenberg, Germany). The collagenase inhibition was calculated using the following equation:

Collagenase inhibition (%) = 1 − (A/B) × 100,(5)

where A and B are the rates of the enzyme reaction of the mixture with and without the sample, respectively. EGCG was used as a positive control. 

#### 3.10.2. Inhibition of the Hyaluronidase Enzyme

*A. mellifera* larval crude proteins and their protein hydrolysates were investigated for their hyaluronidase inhibitory activity using a spectrophotometric assay according to the method of Chaiyana et al. (2020) [65]. Before this study, hyaluronidase from bovine testes was dissolved in 20 mM phosphate buffer (pH 5.35), 0.01% *w*/*v* BSA, and 77 mM NaCl to create a 15 mg/mL hyaluronidase solution. Afterwards, 20 µL of a sample solution with varying concentrations was added to 100 µL of a hyaluronidase solution and incubated at 37 °C for 10 min. Then, 100 µL of 0.03% *w*/*v* hyaluronic acid in phosphate buffer (pH 5.35) was added and incubated at 37 °C for 45 min. After that, 0.1% BSA was added and additionally incubated for 10 min at room temperature. The resulting mixture containing the *A. mellifera* larval crude proteins at a final concentration of 0.1 mg/mL was measured for an absorbance at 600 nm by a microplate reader (BMG Labtech, Ortenberg, Germany). The hyaluronidase inhibitory activity was calculated using the following equation:

Hyaluronidase inhibition (%) = 1 − (A/B) × 100,(6)

where A and B are the absorbance values of the mixture with and without the sample, respectively. Oleanolic acid was used as a positive control. 

### 3.11. Irritation Test by the HET-CAM Test

*A. mellifera* larval crude proteins and their protein hydrolysates were tested for their irritation potential by the HET-CAM assay [66]. The CAM of fertilized hen eggs aged between 7 and 9 days was removed by cutting the aerobic part with a rotating dentist saw blade (Ehwa Technologies Information Co., Ltd., Seoul, Republic of Korea) and gently peeling off. A normal saline solution was dropped in and placed the eggs in the hatching machine (Nanchang Howard Technology Co., Ltd., Jiangxi, China) for 15 min, then the inner layer of the eggshell was removed. Each sample was added and observed for the irritation sign for 5 min and once again after 60 min. The onset of vascular hemorrhage, vascular lysis, and vascular coagulation was recorded, and the irritation score (IS) was calculated using the following equation:

IS = [((301 − H)/300) × 5] + [((301 − L)/300) × 7] + [((301 − C)/300) × 9],(7)

where H is the onset of vascular hemorrhage within 300 s after application (s), L is the onset of vascular lysis within 300 s after application (s), and C is the onset of vascular coagulation within 300 s after application (s). The experiment was repeated three times. The severity of irritation can be interpreted as follows: 0 = no irritation (0.0–0.9), 1 = slight irritation (1.0–4.9), 2 = moderate irritation (5.0–8.9), and 3 = severe irritation (9.0–21.0).

### 3.12. Statistical Analysis

Data were presented as the mean ± standard deviation (S.D.). Differences between samples were analyzed by the one-way ANOVA, followed by Tukey’s post-hoc test using SPSS for Windows (version 17.0, SPSS Inc., Chicago, IL, USA). Statistical significance was considered with *p* < 0.05.

## 4. Conclusions

The present study demonstrates the efficacy of various media used in the crude protein extraction of defatted *A. mellifera* larvae, with the sodium hydroxide extraction showing promising results in terms of its protein content and yield. The subsequent hydrolysis process using the Alcalase^®^ enzyme was proven to be efficient, yielding protein hydrolysates with significant bioactivity. Crude proteins extracted with ascorbic acid and their hydrolysates showed notable inhibitory effects against ABTS^•+^, NO^•^, and collagenase. Remarkably, the inhibitory effects of crude proteins isolated from *A. mellifera* larvae were equivalent to well-known antioxidants and amino acids such as ascorbic acid and lysine. Conversely, crude proteins extracted with sodium hydroxide exhibited remarkable effectiveness in inhibiting hyaluronidase, with the highest inhibitory activity observed at 78.1 ± 1.5%. Moreover, these findings suggested the potential of *A. mellifera* larval proteins as functional ingredients in cosmetics, given their demonstrated safety and efficacy. These findings highlight the diverse bioactive properties of *A. mellifera* larval proteins extracted with different media, suggesting their potential applications across various fields, including pharmaceuticals and cosmetics. Overall, this study contributes valuable insights into the utilization of *A. mellifera* larval proteins as sustainable and multifunctional ingredients with diverse industrial applications. The potential of *A. mellifera* larval proteins as biologically active ingredients in cosmetics and pharmaceuticals highlighted promising industrial applications, particularly in anti-skin wrinkles. Their antioxidant, anti-aging, and low-irritability properties position them as valuable candidates for addressing skincare concerns effectively. Therefore, further development of cosmeceutical formulations and additional research through clinical trials to validate their safety and efficacy were suggested.

## Figures and Tables

**Table 1:** Amino acid profiles of dried and defatted *A. mellifera* larvae.

Amino Acid Profile	Amount (% *w*/*w*)	
	Dried *A. mellifera* Larvae	Defatted *A. mellifera* Larvae
Lysine	9.52 ± 0.18	8.61 ± 0.17
Leucine	4.62 ± 0.02	5.28 ± 0.09
Isoleucine	3.93 ± 0.07	4.33 ± 0.02
Phenylalanine	3.88 ± 0.08	4.54 ± 0.15
Tyrosine	3.00 ± 0.07	3.65 ± 0.25
Glutamic acid	2.18 ± 0.19	2.38 ± 0.01
Valine	2.00 ± 0.02	2.20 ± 0.06
Histidine	1.80 ± 0.01	1.73 ± 0.05
Aspartic acid	1.73 ± 0.02	2.04 ± 0.21
Alanine	1.31 ± 0.13	1.42 ± 0.07
Proline	0.98 ± 0.09	1.08 ± 0.07
Glycine	0.69 ± 0.01	0.76 ± 0.02
Cystine	0.54 ± 0.01	0.68 ± 0.12
Serine	0.53 ± 0.31	0.44 ± 0.13
Threonine	0.44 ± 0.15	0.43 ± 0.07
Tryptophan	0.15 ± 0.00	0.15 ± 0.00
Hydroxylysine	<0.02 ^1^	<0.02 ^1^
Hydroxyproline	<0.02 ^1^	<0.02 ^1^
Methionine	<0.02 ^1^	<0.02 ^1^
Total	37.30	39.72

^1^ The limit of detection was 0.02% *w*/*w*.

**Table 2:** Irritation potentials of dried and defatted *A. mellifera* larvae.

Substance	Irritation Score	Severity of Irritation
Positive control	15.51 ± 0.64 ^a^	Severe irritation
Negative control	0.0 ± 0.0 ^b^	No irritation
Lysine	0.0 ± 0.0 ^b^	No irritation
Crude proteins		
● SHE	0.0 ± 0.0 ^b^	No irritation
● WTE	0.0 ± 0.0 ^b^	No irritation
● AAE	0.0 ± 0.0 ^b^	No irritation
● CAE	0.0 ± 0.0 ^b^	No irritation
● HCE	0.0 ± 0.0 ^b^	No irritation
Protein hydrolysate		
● SHE	0.0 ± 0.0 ^b^	No irritation
● WTE	0.0 ± 0.0 ^b^	No irritation
● AAE	0.0 ± 0.0 ^b^	No irritation
● CAE	0.0 ± 0.0 ^b^	No irritation
● HCE	0.0 ± 0.0 ^b^	No irritation

NOTE: Data are expressed as the mean ± S.D (*n* = 2). The 1% *w*/*v* sodium lauryl sulfate (SLS) was used as a positive control; normal saline solution (NSS) was used as a negative control; and lysine, a major amino acid detected in *A. mellifera* larvae, was also tested in a comparison with crude proteins and their hydrolysates extracted using various media, including sodium hydroxide (SHE), DI water (WTE), ascorbic acid (AAE), citric acid (CAE), and hydrochloric acid (HCE). Lowercase letters (a,b) indicate significant differences between the *A. mellifera* larval extracts. The identical letters represent values that do not exhibit statistically significant differences. The data were analyzed using a one-way ANOVA, followed by Tukey’s post-hoc test (*p* < 0.05).

## Data Availability

The data presented in this study are available on request from the corresponding author.

## Supplementary Materials

The following supporting information can be downloaded at: https://www.mdpi.com/article/10.3390/ph17060679/s1, Table S1: The *p*-values demonstrate statistically significant differences (*p* < 0.05, labeled in red) in yields among defatted *A. mellifera* larvae, as well as in their crude protein extracts and protein hydrolysates; Table S2: Tukey’s honestly significant difference (HSD) test results (α = 0.05) for yields among defatted *A. mellifera* larvae, crude protein extracts, and protein hydrolysates; Table S3: The *p*-values demonstrate statistically significant differences (*p* < 0.05, labeled in red) in total protein contents among crude protein extracts and protein hydrolysates of *A. mellifera* larvae; Table S4: Tukey’s HSD test results (α = 0.05) for total protein contents among crude protein extracts and protein hydrolysates of *A. mellifera* larvae; Table S5: Antioxidant activities of *A. mellifera* larval extracts; Table S6: The *p*-values demonstrate statistically significant differences (*p* < 0.05, labeled in red) in Trolox equivalent antioxidant capacity (TEAC) among crude protein extracts and protein hydrolysates of *A. mellifera* larvae; Table S7: Tukey’s HSD test results (α = 0.05) for TEAC among crude protein extracts and protein hydrolysates of *A. mellifera* larvae; Table S8: The *p*-values demonstrate statistically significant differences (*p* < 0.05, labeled in red) in nitric oxide (NO^•^) inhibition among crude protein extracts and protein hydrolysates of *A. mellifera* larvae; Table S9: Tukey’s HSD test results (α = 0.05) for NO^•^ inhibition among crude protein extracts and protein hydrolysates of *A. mellifera* larvae; Table S10: Anti-skin aging activities of *A. mellifera* larval extracts; Table S11: The *p*-values demonstrate statistically significant differences (*p* < 0.05, labeled in red) in collagenase inhibition among crude protein extracts and protein hydrolysates of *A. mellifera* larvae; Table S12: Tukey’s HSD test results (α = 0.05) for collagenase inhibition among crude protein extracts and protein hydrolysates of *A. mellifera* larvae; Table S13: The *p*-values demonstrate statistically significant differences (*p* < 0.05, labeled in red) in hyaluronidase inhibition among crude protein extracts and protein hydrolysates of *A. mellifera* larvae; Table S14: Tukey’s HSD test results (α = 0.05) for hyaluronidase inhibition among crude protein extracts and protein hydrolysates of *A. mellifera* larvae.
