# Supplementary material for: Potential of Bioactive Protein and Protein Hydrolysate from Apis mellifera Larvae as Cosmeceutical Active Ingredients for Anti-Skin Aging
Source: Pharmaceuticals (Basel). 2024 May 24;17(6):679. doi: 10.3390/ph17060679 (PMC11206733; doi:10.3390/ph17060679)
Supplement: Supplementary file 1 [file pharmaceuticals-17-00679-s001.zip › pharmaceuticals-2918454-supplementary (1).pdf]

pharmaceuticals

Supplementary Materials

Potential of Bioactive Protein and Protein Hydrolysate from *Apis mellifera* Larvae as Cosmeceutical Active Ingredients for Anti-Skin Aging

Paphawarin Thuraphan [1], Anurak Bunrod [2], Watchara Kanjanakawinkul [2] and Wantida Chaiyana [1,3,4,*]

[1] Department of Pharmaceutical Sciences, Faculty of Pharmacy, Chiang Mai University, Chiang Mai 50200, Thailand; paphawarin_thu@cmu.ac.th (P.T.)
[2] Chulabhorn Royal Pharmaceutical Manufacturing Facilities by Chulabhorn Royal Academy, Chon Buri 20180, Thailand; anurak.bun@cra.ac.th (A.B.); watchara.kan@cra.ac.th (W.K.)
[3] Center of Excellence in Pharmaceutical Nanotechnology, Faculty of Pharmacy, Chiang Mai University, Chiang Mai 50200, Thailand
[4] Multidisciplinary and Interdisciplinary School, Chiang Mai University, Chiang Mai 50200, Thailand
* Correspondence: wantida.chaiyana@cmu.ac.th; Tel.: +6653944343

Table S1. The p-values demonstrate statistically significant differences ($p < 0.05$, labeled in red) in yields among defatted *A. mellifera* larvae, as well as in their crude protein extracts and protein hydrolysates.

Samples		Group	DL	SHE		WTE		AAE		CAE		HCE	
				CP	PH	CP	PH	CP	PH	CP	PH	CP	PH
DL		a		0.004	0.000	0.000	0.000	0.003	0.002	0.000	0.000	0.000	0.000
SHE	CP	b	0.004		0.613	0.003	0.002	1.000	1.000	0.997	0.988	0.067	0.018
	PH	b,c,d	0.000	0.613		0.233	0.170	0.680	0.759	0.982	0.995	0.944	0.665
WTE	CP	d	0.000	0.003	0.233		1.000	0.004	0.005	0.024	0.035	0.936	0.999
	PH	d	0.000	0.002	0.170	1.000		0.003	0.004	0.016	0.023	0.874	0.995
AAE	CP	b	0.003	1.000	0.680	0.004	0.003		1.000	0.999	0.995	0.084	0.023
	PH	b	0.002	1.000	0.759	0.005	0.004	1.000		1.000	0.998	0.110	0.031
CAE	CP	b,c	0.000	0.997	0.982	0.024	0.016	0.999	1.000		1.000	0.345	0.123
	PH	b,c	0.000	0.988	0.995	0.035	0.023	0.995	0.998	1.000		0.440	0.169
HCE	CP	b,c,d	0.000	0.067	0.944	0.936	0.874	0.084	0.110	0.345	0.440		1.000
	PH	c,d	0.000	0.018	0.665	0.999	0.995	0.023	0.031	0.123	0.169	1.000	

Note: DL = defatted *A. mellifera* larvae; CP = crude proteins; PH = proteins hydrolysates; PHs were obtained from CPs extracted using various media, including sodium hydroxide (SHE), DI water (WTE), ascorbic acid (AAE), citric acid (CAE), and hydrochloric acid (HCE). The identical letters (a, b, c, and d) represent values that do not exhibit statistically significant differences among *A. mellifera* larvae extracts, while differing letters indicate significant differences. The data were analyzed using One-Way ANOVA followed by Tukey's post-hoc test ($p < 0.05$).

Table S2. Tukey's honestly significant difference (HSD) test results ($\alpha = 0.05$) for yields among defatted *A. mellifera* larvae, crude protein extracts, and protein hydrolysates.

Samples	Subset for alpha = 0.05				Group
	a	b	c	d	
DL	78.1433				a
SHE-CP		48.7458			b
AAE-CP		48.0669			b
AAE-PH		47.2338			b
CAE-CP		43.1178	43.1178		b,c
CAE-PH		42.0457	42.0457		b,c
SHE-PH		36.0181	36.0181	36.0181	b,c,d
HCE-CP		27.5665	27.5665	27.5665	b,c,d
HCE-PH			23.8157	23.8157	c,d
WTE-CP				18.9331	d
WTE-PH				17.8022	d

Note: DL = defatted *A. mellifera* larvae; CP = crude proteins; PH = proteins hydrolysates; PHs were obtained from CPs extracted using various media, including sodium hydroxide (SHE), DI water (WTE), ascorbic acid (AAE), citric acid (CAE), and hydrochloric acid (HCE). The identical letters (a, b, c, and d) represent values that do not exhibit statistically significant differences among *A. mellifera* larvae extracts, while differing letters indicate significant differences. The data were analyzed using One-Way ANOVA followed by Tukey's post-hoc test ($p < 0.05$).

Table S3. The p-values demonstrate statistically significant differences ($p < 0.05$, labeled in red) in total protein contents among crude protein extracts and protein hydrolysates of *A. mellifera* larvae.

Samples		Group	SHE		WTE		AAE		CAE		HCE	
			CP	PH	CP	PH	CP	PH	CP	PH	CP	PH
SHE	CP	a		0.000	0.000	0.000	0.000	0.000	0.000	0.000	0.000	0.000
	PH	b	0.000		1.000	0.000	0.003	0.000	0.096	0.000	0.874	0.000
WTE	CP	b	0.000	1.000		0.000	0.011	0.000	0.246	0.000	0.990	0.000
	PH	e	0.000	0.000	0.000		0.000	1.000	0.000	0.080	0.000	0.000
AAE	CP	c	0.000	0.003	0.011	0.000		0.000	0.841	0.000	0.083	0.000
	PH	e	0.000	0.000	0.000	1.000	0.000		0.000	0.181	0.000	0.000
CAE	CP	b,c	0.000	0.096	0.246	0.000	0.841	0.000		0.000	0.783	0.000
	PH	e	0.000	0.000	0.000	0.080	0.000	0.181	0.000		0.000	0.000
HCE	CP	b,c	0.000	0.874	0.990	0.000	0.083	0.000	0.783	0.000		0.000
	PH	d	0.000	0.000	0.000	0.000	0.000	0.000	0.000	0.000	0.000	

Note: CP = crude proteins; PH = proteins hydrolysates; PHs were obtained from CPs extracted using various media, including sodium hydroxide (SHE), DI water (WTE), ascorbic acid (AAE), citric acid (CAE), and hydrochloric acid (HCE). The identical letters (a, b, c, d, and e) represent values that do not exhibit statistically significant differences among *A. mellifera* larvae extracts, while differing letters indicate significant differences. The data were analyzed using One-Way ANOVA followed by Tukey's post-hoc test ($p < 0.05$).

Table S4. Tukey's HSD test results ($\alpha = 0.05$) for total protein contents among crude protein extracts and protein hydrolysates of *A. mellifera* larvae.

Samples	Subset for alpha = 0.05					Group
	a	b	c	d	e	
SHE-CP	173.1030					a
SHE-HP		6.2087				b
WTE-CP		6.0976				b
HCE-CP		5.8915	5.8915			b,c
CAE-CP		5.5339	5.5339			b,c
AAE-CP			5.2006			c
HCE-HP				3.1501		d
WTE-HP					0.8026	e
AAE-HP					0.7097	e
CAE-HP					0.1077	e

Note: CP = crude proteins; PH = proteins hydrolysates; PHs were obtained from CPs extracted using various media, including sodium hydroxide (SHE), DI water (WTE), ascorbic acid (AAE), citric acid (CAE), and hydrochloric acid (HCE). The identical letters (a, b, c, d, and e) represent values that do not exhibit statistically significant differences among *A. mellifera* larvae extracts, while differing letters indicate significant differences. The data were analyzed using One-Way ANOVA followed by Tukey's post-hoc test ($p < 0.05$).

Table S5. Antioxidant activities of *A. mellifera* larval extracts.

Samples	TEAC (μg Trolox/mg sample)		NO• inhibition (%)	
GA	N/A		73.63 ± 1.46^{b}	
ASC	36.65 ± 0.11^{a}		89.79 ± 2.57^{a}	
LYS	$31.97 \pm 0.67^{b,c}$		$82.43 \pm 1.57^{a,b}$	
***A. mellifera* larval extract**	**Crude protein**	**Protein hydrolysate**	**Crude protein**	**Protein hydrolysate**
SHE	$29.06 \pm 0.99^{c,d,e}$	$34.64 \pm 0.27^{a,b}$	10.11 ± 4.23^{e}	$15.20 \pm 4.02^{d,e}$
WTE	$29.50 \pm 2.71^{c,d}$	$31.89 \pm 0.05^{b,c}$	25.01 ± 1.48^{d}	$20.59 \pm 5.36^{d,e}$
AAE	$33.81 \pm 1.15^{a,b}$	$34.02 \pm 0.24^{a,b}$	51.98 ± 9.88^{c}	43.84 ± 5.16^{c}
CAE	25.95 ± 2.25^{e}	$32.00 \pm 1.05^{b,c}$	$14.91 \pm 2.53^{d,e}$	11.48 ± 2.66^{e}
HCE	$29.26 \pm 0.67^{c,d,e}$	$28.23 \pm 0.82^{d,e}$	$20.48 \pm 5.04^{d,e}$	$13.08 \pm 3.43^{d,e}$

Note: TEAC = Trolox equivalent antioxidant capacity; NO• = nitric oxide; GA = gallic acid; ASC = ascorbic acid; LYS = lysine; crude proteins and their hydrolysates extracted using various media, including sodium hydroxide (SHE), DI water (WTE), ascorbic acid (AAE), citric acid (CAE), and hydrochloric acid (HCE); N/A = not available. Lowercase letters (a, b, c, d, and e) indicate significant differences among *A. mellifera* larvae extracts. The identical letters represent values that do not exhibit statistically significant differences. The data were analyzed using One-Way ANOVA followed by Tukey's post-hoc test ($p < 0.05$).

Table S6. The p-values demonstrate statistically significant differences ($p < 0.05$, labeled in red) in Trolox equivalent antioxidant capacity (TEAC) among crude protein extracts and protein hydrolysates of *A. mellifera* larvae.

Samples	Group	ASC	LYS	SHE	WTE	AAE	CAE	HCE

					CP	PH	CP	PH	CP	PH	CP	PH	CP	PH
**ASC**		a		0.004	0.000	0.661	0.000	0.003	0.207	0.294	0.000	0.004	0.000	0.000
**LYS**		b,c	0.004		0.181	0.281	0.379	1.000	0.768	0.643	0.000	1.000	0.261	0.032
**SHE**	**CP**	c,d,e	0.000	0.181		0.000	1.000	0.212	0.003	0.002	0.125	0.171	1.000	0.999
	**PH**	a,b	0.661	0.281	0.000		0.001	0.242	0.999	1.000	0.000	0.294	0.001	0.000
**WTE**	**CP**	c,d	0.000	0.379	1.000	0.001		0.429	0.009	0.005	0.050	0.362	1.000	0.972
	**PH**	b,c	0.003	1.000	0.212	0.242	0.429		0.716	0.586	0.000	1.000	0.302	0.039
**AAE**	**CP**	a,b	0.207	0.768	0.003	0.999	0.009	0.716		1.000	0.000	0.785	0.005	0.000
	**PH**	a,b	0.294	0.643	0.002	1.000	0.005	0.586	1.000		0.000	0.661	0.003	0.000
**CAE**	**CP**	e	0.000	0.000	0.125	0.000	0.050	0.000	0.000	0.000		0.000	0.082	0.492
	**PH**	b,c	0.004	1.000	0.171	0.294	0.362	1.000	0.785	0.661	0.000		0.248	0.030
**HCE**	**CP**	c,d,e	0.000	0.261	1.000	0.001	1.000	0.302	0.005	0.003	0.082	0.248		0.994
	**PH**	d,e	0.000	0.032	0.999	0.000	0.972	0.039	0.000	0.000	0.492	0.030	0.994	

Note: ASC = ascorbic acid; LYS = lysine; CP = crude proteins; PH = proteins hydrolysates; PHs were obtained from CPs extracted using various media, including sodium hydroxide (SHE), DI water (WTE), ascorbic acid (AAE), citric acid (CAE), and hydrochloric acid (HCE). The identical letters (a, b, c, d, and e) represent values that do not exhibit statistically significant differences among *A. mellifera* larvae extracts, while differing letters indicate significant differences. The data were analyzed using One-Way ANOVA followed by Tukey's post-hoc test ($p < 0.05$).

Table S7. Tukey's HSD test results ($\alpha = 0.05$) for TEAC among crude protein extracts and protein hydrolysates of *A. mellifera* larvae.

**Samples**	**Subset for alpha = 0.05**					**Group**
	**a**	**b**	**c**	**d**	**e**	
ASC	36.6563					a
SHE-PH	34.6415	34.6415				a,b
AAE-PH	34.0193	34.0193				a,b
AAE-CP	33.8119	33.8119				a,b
CAE-PH		32.0044	32.0044			b,c
LYS		31.9748	31.9748			b,c
WTE-PH		31.8859	31.8859			b,c
WTE-CP			29.5007	29.5007		c,d
HCE-CP			29.2637	29.2637	29.2637	c,d,e
SHE-CP			29.0563	29.0563	29.0563	c,d,e
HCE-PH				28.2267	28.2267	d,e
CAE-CP					25.9452	e

Note: ASC = ascorbic acid; LYS = lysine; CP = crude proteins; PH = proteins hydrolysates; PHs were obtained from CPs extracted using various media, including sodium hydroxide (SHE), DI water (WTE), ascorbic acid (AAE), citric acid (CAE), and hydrochloric acid (HCE). The identical letters (a, b, c, d, and e) represent values that do not exhibit statistically significant differences among *A. mellifera* larvae extracts, while differing letters indicate significant differences. The data were analyzed using One-Way ANOVA followed by Tukey's post-hoc test ($p < 0.05$).

Table S8. The p-values demonstrate statistically significant differences ($p < 0.05$, labeled in red) in nitric oxide ($NO^{\bullet}$) inhibition among crude protein extracts and protein hydrolysates of *A. mellifera* larvae.

**Samples**		**Group**	**GA**	**ASC**	**LYS**	**SHE**		**WTE**		**AAE**		**CAE**		**HCE**	
						**CP**	**PH**	**CP**	**PH**	**CP**	**PH**	**CP**	**PH**	**CP**	**PH**
**GA**		b		0.001	0.088	0.000	0.000	0.000	0.000	0.000	0.000	0.000	0.000	0.000	0.000
**ASC**		a	0.001		0.693	0.000	0.000	0.000	0.000	0.000	0.000	0.000	0.000	0.000	0.000
**LYS**		a,b	0.088	0.693		0.000	0.000	0.000	0.000	0.000	0.000	0.000	0.000	0.000	0.000
**SHE**	**CP**	e	0.000	0.000	0.000		0.962	0.015	0.212	0.000	0.000	0.975	1.000	0.224	1.000
	**PH**	d,e	0.000	0.000	0.000	0.962		0.290	0.943	0.000	0.000	1.000	0.997	0.950	1.000
**WTE**	**CP**	d	0.000	0.000	0.000	0.015	0.290		0.987	0.000	0.001	0.255	0.037	0.984	0.097
	**PH**	d,e	0.000	0.000	0.000	0.212	0.943	0.987		0.000	0.000	0.921	0.393	1.000	0.668
**AAE**	**CP**	c	0.001	0.000	0.000	0.000	0.000	0.000	0.000		0.557	0.000	0.000	0.000	0.000
	**PH**	c	0.000	0.000	0.000	0.000	0.000	0.001	0.000	0.557		0.000	0.000	0.000	0.000
**CAE**	**CP**	d,e	0.000	0.000	0.000	0.975	1.000	0.255	0.921	0.000	0.000		0.999	0.930	1.000
	**PH**	e	0.000	0.000	0.000	1.000	0.997	0.037	0.393	0.000	0.000	0.999		0.410	1.000
**HCE**	**CP**	d,e	0.000	0.000	0.000	0.224	0.950	0.984	1.000	0.000	0.000	0.930	0.410		0.687
	**PH**	d,e	0.000	0.000	0.000	1.000	1.000	0.097	0.668	0.000	0.000	1.000	1.000	0.687	

Note: GA = gallic acid; ASC = ascorbic acid; LYS = lysine; CP = crude proteins; PH = proteins hydrolysates; PHs were obtained from CPs extracted using various media, including sodium hydroxide (SHE), DI water (WTE), ascorbic acid (AAE), citric acid (CAE), and hydrochloric acid (HCE). The identical letters (a, b, c, d, and e) represent values that do not exhibit statistically significant differences among *A. mellifera* larvae extracts, while differing letters indicate significant differences. The data were analyzed using One-Way ANOVA followed by Tukey's post-hoc test ($p < 0.05$).

Table S9. Tukey's HSD test results ($\alpha = 0.05$) for $NO^{\bullet}$ inhibition among crude protein extracts and protein hydrolysates of *A. mellifera* larvae.

**Samples**	**Subset for alpha = 0.05**					**Group**
	**a**	**b**	**c**	**d**	**e**	

ASC	89.7935					a
LYS	82.4303	82.4303				a,b
GA		70.3335				b
AAE-CP			51.9850			c
AAE-PH			43.8439			c
WTE-CP				25.0134		d
WTE-PH				20.5874	20.5874	d,e
HCE-CP				20.4802	20.4802	d,e
SHE-PH				15.1958	15.1958	d,e
CAE-CP				14.9142	14.9142	d,e
HCE-PH				13.0767	13.0767	d,e
CAE-PH					11.4807	e
SHE-CP					10.1127	e

Note: GA = gallic acid; ASC = ascorbic acid; LYS = lysine; CP = crude proteins; PH = proteins hydrolysates; PHs were obtained from CPs extracted using various media, including sodium hydroxide (SHE), DI water (WTE), ascorbic acid (AAE), citric acid (CAE), and hydrochloric acid (HCE). The identical letters (a, b, c, d, and e) represent values that do not exhibit statistically significant differences among *A. mellifera* larvae extracts, while differing letters indicate significant differences. The data were analyzed using One-Way ANOVA followed by Tukey's post-hoc test ($p < 0.05$).

Table S10. Anti-skin ageing activities of *A. mellifera* larval extracts.

Samples	Collagenase inhibition (%)		Hyaluronidase inhibition (%)	
EGCG	75.74 ± 4.98^{a}		N/A	
OA	N/A		85.66 ± 6.59^{a}	
ASC	31.15 ± 10.81^{b,c,d,e}		0.00 ± 3.65^{h}	
LYS	39.86 ± 9.84^{b,c,d}		4.48 ± 2.95^{g}	
***A. mellifera* larval extract**	**Crude protein**	**Protein hydrolysate**	**Crude protein**	**Protein hydrolysate**
SHE	12.54 ± 1.182^{e,f}	14.82 ± 2.22^{d,e,f}	78.15 ± 1.55^{a}	13.79 ± 4.32^{e,f,g}
WTE	0.00 ± 3.28^{f}	34.00 ± 8.20^{b,c,d,e}	56.40 ± 1.36^{b}	37.58 ± 5.37^{c}
AAE	48.11 ± 14.26^{b}	44.54 ± 1.34^{b,c}	27.54 ± 2.27^{c,d}	4.09 ± 1.86^{g}
CAE	32.11 ± 8.04^{b,c,d,e}	2.51 ± 0.24^{f}	18.39 ± 5.18^{d,e,f}	14.60 ± 2.08^{e,f,g}
HCE	18.67 ± 1.02^{c,d,e,f}	14.85 ± 4.07^{d,e,f}	24.24 ± 5.11^{d,e}	8.68 ± 1.36^{f,g}

Note: EGCG = epigallocatechin gallate; OA = oleanolic acid; ASC = ascorbic acid; LYS = lysine; crude proteins and their hydrolysates extracted using various media, including sodium hydroxide (SHE), DI water (WTE), ascorbic acid (AAE), citric acid (CAE), and hydrochloric acid (HCE); N/A = not available. Lowercase letters (a, b, c, d, e, f, g, and h) indicate significant differences among *A. mellifera* larvae extracts. The identical letters represent values that do not exhibit statistically significant differences. The data were analyzed using One-Way ANOVA followed by Tukey's post-hoc test ($p < 0.05$).

Table S11. The p-values demonstrate statistically significant differences ($p < 0.05$, labeled in red) in collagenase inhibition among crude protein extracts and protein hydrolysates of *A. mellifera* larvae.

Samples		Group	EGCG	ASC	LYS	SHE		WTE		AAE		CAE		HCE	
						CP	PH	CP	PH	CP	PH	CP	PH	CP	PH
**EGCG**		a		0.001	0.006	0.000	0.000	0.000	0.002	0.046	0.019	0.001	0.000	0.000	0.000
**ASC**		b,c,d,e	0.001		0.977	0.339	0.504	0.020	1.000	0.454	0.743	1.000	0.036	0.811	0.506
**LYS**		b,c,d	0.006	0.977		0.049	0.084	0.002	0.999	0.985	1.000	0.991	0.004	0.201	0.085
**SHE**	**CP**	e,f	0.000	0.339	0.049		1.000	0.806	0.190	0.007	0.016	0.281	0.941	0.999	1.000
	**PH**	d,e,f	0.000	0.504	0.084	1.000		0.628	0.303	0.012	0.028	0.430	0.822	1.000	1.000
**WTE**	**CP**	f	0.000	0.020	0.002	0.806	0.628		0.010	0.000	0.001	0.016	1.000	0.335	0.625
	**PH**	b,c,d,e	0.002	1.000	0.999	0.190	0.303	0.010		0.686	0.922	1.000	0.018	0.585	0.305
**AAE**	**CP**	b	0.046	0.454	0.985	0.007	0.012	0.000	0.686		1.000	0.530	0.001	0.030	0.012
	**PH**	b,c	0.019	0.743	1.000	0.016	0.028	0.001	0.922	1.000		0.814	0.002	0.070	0.028
**CAE**	**CP**	b,c,d,e	0.001	1.000	0.991	0.281	0.430	0.016	1.000	0.530	0.814		0.028	0.740	0.432
	**PH**	f	0.000	0.036	0.004	0.941	0.822	1.000	0.018	0.001	0.002	0.028		0.517	0.820
**HCE**	**CP**	c,d,e,f	0.000	0.811	0.201	0.999	1.000	0.335	0.585	0.030	0.070	0.740	0.517		1.000
	**PH**	d,e,f	0.000	0.506	0.085	1.000	1.000	0.625	0.305	0.012	0.028	0.432	0.820	1.000	

Note: EGCG = epigallocatechin gallate; ASC = ascorbic acid; LYS = lysine; CP = crude proteins; PH = proteins hydrolysates; PHs were obtained from CPs extracted using various media, including sodium hydroxide (SHE), DI water (WTE), ascorbic acid (AAE), citric acid (CAE), and hydrochloric acid (HCE). The identical letters (a, b, c, d, e, and f) represent values that do not exhibit statistically significant differences among *A. mellifera* larvae extracts, while differing letters indicate significant differences. The data were analyzed using One-Way ANOVA followed by Tukey's post-hoc test ($p < 0.05$).

Table S12. Tukey's HSD test results ($\alpha = 0.05$) for collagenase inhibition among crude protein extracts and protein hydrolysates of *A. mellifera* larvae.

Samples	Subset for alpha = 0.05						Group
	a	b	c	d	e	f	

EGCG	75.7432						a
AAE-CP		48.1141					b
AAE-PH		44.5373	44.5373				b,c
LYS		39.8644	39.8644	39.8644			b,c,d
WTE-PH		34.0022	34.0022	34.0022	34.0022		b,c,d,e
CAE-CP		32.1070	32.1070	32.1070	32.1070		b,c,d,e
ASC		31.1501	31.1501	31.1501	31.1501		b,c,d,e
HCE-CP			18.6734	18.6734	18.6734	18.6734	c,d,e,f
HCE-PH				14.8458	14.8458	14.8458	d,e,f
SHE-PH				14.8180	14.8180	14.8180	d,e,f
SHE-CP					12.5416	12.5416	e,f
CAE-PH						2.5084	f
WTE-CP						0.0000	f

Note: EGCG = epigallocatechin gallate; ASC = ascorbic acid; LYS = lysine; CP = crude proteins; PH = proteins hydrolysates; PHs were obtained from CPs extracted using various media, including sodium hydroxide (SHE), DI water (WTE), ascorbic acid (AAE), citric acid (CAE), and hydrochloric acid (HCE). The identical letters (a, b, c, d, e, and f) represent values that do not exhibit statistically significant differences among *A. mellifera* larvae extracts, while differing letters indicate significant differences. The data were analyzed using One-Way ANOVA followed by Tukey's post-hoc test ($p < 0.05$).

Table S13. The p-values demonstrate statistically significant differences ($p < 0.05$, labeled in red) in hyaluronidase inhibition among crude protein extracts and protein hydrolysates of *A. mellifera* larvae.

Samples		Group	OA	ASC	LYS	SHE		WTE		AAE		CAE		HCE	
						CP	PH	CP	PH	CP	PH	CP	PH	CP	PH
**OA**		a		0.000	0.000	0.573	0.000	0.000	0.000	0.000	0.000	0.000	0.000	0.000	0.000
**ASC**		h	0.000		0.011	0.000	0.000	0.000	0.000	0.000	0.040	0.000	0.000	0.000	0.000
**LYS**		g	0.000	0.011		0.000	0.154	0.000	0.000	0.000	1.000	0.005	0.091	0.000	0.964
**SHE**	**CP**	a	0.573	0.000	0.000		0.000	0.000	0.000	0.000	0.000	0.000	0.000	0.000	0.000
	**PH**	e,f,g	0.000	0.000	0.154	0.000		0.000	0.000	0.006	0.225	0.932	1.000	0.072	0.874
**WTE**	**CP**	b	0.000	0.000	0.000	0.000	0.000		0.000	0.000	0.000	0.000	0.000	0.000	0.000
	**PH**	c	0.000	0.000	0.000	0.000	0.000	0.000		0.096	0.000	0.000	0.000	0.008	0.000
**AAE**	**CP**	c,d	0.000	0.000	0.000	0.000	0.006	0.000	0.096		0.000	0.170	0.011	0.995	0.000
	**PH**	g	0.000	0.000	1.000	0.000	0.225	0.000	0.000	0.000		0.013	0.145	0.000	0.969
**CAE**	**CP**	d,e,f	0.000	0.000	0.005	0.000	0.932	0.000	0.000	0.170	0.013		0.028	0.740	0.432
	**PH**	e,f,g	0.000	0.000	0.091	0.000	1.000	0.000	0.000	0.011	0.145	0.028		0.517	0.820
**HCE**	**CP**	d,e	0.000	0.000	0.000	0.000	0.072	0.000	0.008	0.995	0.000	0.740	0.517		1.000
	**PH**	f,g	0.000	0.000	0.964	0.000	0.874	0.000	0.000	0.000	0.969	0.432	0.820	1.000	

Note: OA = oleanolic acid; ASC = ascorbic acid; LYS = lysine; CP = crude proteins; PH = proteins hydrolysates; PHs were obtained from CPs extracted using various media, including sodium hydroxide (SHE), DI water (WTE), ascorbic acid (AAE), citric acid (CAE), and hydrochloric acid (HCE). The identical letters (a, b, c, d, e, f, g, and h) represent values that do not exhibit statistically significant differences among *A. mellifera* larvae extracts, while differing letters indicate significant differences. The data were analyzed using One-Way ANOVA followed by Tukey's post-hoc test ($p < 0.05$).

Table S14. Tukey's HSD test results ($\alpha = 0.05$) for hyaluronidase inhibition among crude protein extracts and protein hydrolysates of *A. mellifera* larvae.

Samples	Subset for alpha = 0.05								Group
	a	b	c	d	e	f	g	h	
OA	85.6566								a
SHE-CP	78.1451								a
WTE-CP		56.4032							b
WTE-PH			37.5762						c
AAE-CP			27.5384	27.5384					c,d
HCE-CP				24.2360	24.2360				d,e
CAE-CP				18.3922	18.3922	18.3922			d,e,f
CAE-PH					14.6003	14.6003	14.6003		e,f,g
SHE-PH					13.7870	13.7870	13.7870		e,f,g
HCE-PH						8.6820	8.6820		f,g
LYS							4.4834		g
AAE-PH							4.0916		g
ASC								0.0000	h

Note: OA = oleanolic acid; ASC = ascorbic acid; LYS = lysine; CP = crude proteins; PH = proteins hydrolysates; PHs were obtained from CPs extracted using various media, including sodium hydroxide (SHE), DI water (WTE), ascorbic acid (AAE), citric acid (CAE), and hydrochloric acid (HCE). The identical letters (a, b, c, d, e, f, g, and h) represent values that do not exhibit statistically significant differences among *A. mellifera* larvae extracts, while differing letters indicate significant differences. The data were analyzed using One-Way ANOVA followed by Tukey's post-hoc test ($p < 0.05$).
